# Eighth Annual Meeting of the American Medical Association

**Published:** 1855-07

**Authors:** 


### EDITORIAL DEPARTMENT.

The following is a part of the proccedings of A. M. A., and will conclude the report in the next number. We call the attention of the reader to the eloquent and masterly effort of Mayor Conrad in reply to an able address of Dr. Hays. It would be a source of profound gratification if all would do the justice he has been so generous as to accord to the profession. Amid our trials and difficulties we wonld then find consolation in the assurance that our labors and sacrifices were appreciated.—Ed. Jour.

Eighth Annual Meeting of the American Medical Association.—This body met on Tuesday, May 1, at Musical Fnnd Hall, in Philadelphia. During Monday a large number of delegates arrived, and there was a full attendanee at the Hall on Tuesday, A. M. The Hall had been fitted up for the convention. In the lower room was placed a finely executed stone intended for the Washington Monument. The stone bears the following inscription.

INSTITUTED

MDCCCXLVII.

Vincit Amor Patriæ.

On the face of the stone was cut, in alto relievo, a most beautiful representation of Hippocrates refusing a bribe tendered him by Artaxerxes, the King of Persia. The stone is four feet by two, and twenty inches in thickness.

The Hall is divided into two portions, one for the convention, and the other for the persons who have tickcts of admission to witness the proceedings. At an early hour, a large number of the delegates presented their credentials in the lower room, and their names were duly registered. A committee continued to sit there for the reception of delegates from abroad.

The following officers of the association appeared in their seats:

*President*—Dr. CHAS. A. POPE, of St. Lonis.

*Secretaries*—Dr. Francis West, of Philadelphia, and Dr. E. S. Lemoine, of St. Louis.

The Association was not called to order until half past eleven o'clock.

The President invited all ex-Presidents and ex-Vicc-Presidents to take seats on the platform.

The first business in order was the report of the committee of arrangement.

Dr. Isaac Hays, of Philadelphia, Chairman of the Committee of Arrangements, said that on behalf of the profession of Philadelphia, he would extend a cordial greeting to the members of the Association. It gave them the highest pleasure to have the Association among them. The committee had made the best arrangements in their power, to render the sojourn of the delegates agreeable. While this was felt as a duty, it was also a gratification.—Eight years have elapsed since this Association was first organized in Philadelphia. In every city in which the annual meetings of the society have been held, our delegates have been treated with an elegant hospitality which we cannot rival. But it was hoped the efforts of the committee would render agreeable the stay of the assembled wisdom of the noblest of professions. Dr. Hays then stated that 337 delegates had registered their names. By general consent the usual calling over of this long list of names would be dispensed with, and the association proceed to the next order of business, which was the calling of the roll.

Dr. West then called the roll.

The address of the President, Dr. Charles A. Pope, of Missouri, was read after the calling of the roll. We give it in full.

GENTLEMEN—With feelings of grateful pleasure, I meet you, and greet you on this occasion.

For high and useful purposes have we assembled from the wide extent of our beloved country. The elevation of a noble profession—the promotion of science—the good of humanity—these have been, are, and will continue to be the objects of our association.—Whether we have, thus far, done much or little, our sole aim has been the advancement of the best interests of our fellow men. I shall not assert that we have done as much as we might have done, or that the course hitherto pursued by us is so perfect as to admit of no improvement. Were such the fact, and were the association a firmly established institution, I might have experienced more hesitation in the selection of a theme for the present occasion. And since we cannot, as yet, I think, urge such a claim, the few suggestions which I shall offer, are made with becoming diffidence, but at the same time with a deep sense of their importance to the welfare and perpetuity of our association.

Some strictures on our proceedings, in medical and other journals, have appeared within the last year, as well as in previous years. I shall not here blame the authors of them. They are, doubtless, as proud of our noble profession as we, and equally with us, anxious for the advancement of its interests and its honor. I thank them for their suggestions. All of us are ready to hear them and to profit by them. If any more effectual mode of arriving at truth can be devised than that which we have heretofore pursued, all of us are ready to follow it, and would rather thank than quarrel with those who propose it.

Physicians have an almost superhuman mission to fulfil. The goal of their ambition, and their hopes, and their duty, stands at the *ultima thule* of human capacity—nay, rather beyond it. It cannot, indeed, be said that their duties are beyond their powers, but their ambition, their hopes, their wishes certainly are. They would gladly know, not only all the secrets of organization, but those also of Pathology and Therapeutics. To arrive at such knowledge is, perhaps, beyond the attainment of the human mind. Multiform are the elements which enter into the problem of health and disease. Health is, itself, a constant change of composition—diseases are ever varying changes, supervening on this.

Do we know, with all our advancement, and after all the toil of our predecessors for two thousand years, the exact changes in which any disease, the fevers, for instance, consists? And even when we shall have learned these, so as to understand them as well as the most ordinary chemical changes, the ever varying character of most diseases, and the inward disturbing influences upon them of the mental an moral emotions, would require to follow them, a continued stretch and power of intellect, of which it is doubtful if man be capable. This exactness of knowledge is not, I grant, necessary to the very successful practice of medicine. Our profession can render great and important services to man without it, but with it, it would be still more serviceable. To it our ambition tends. To this perfect knowledge we aspire. Although we may never reach, we can yet eternally approach it. In the vast region of our researches, there is no probability that human genius will ever, Alexander-like, weep for the want of unconquered provinces. Beyond the conquests of the future heroes of the profession, there will always be a boundless field for the ambitious and philanthropic explorer. In the language of a western student, "the science of Medicine, like the liver of Prometheus, is sufficient to glut the eagles of all time."

The object of this association is to do something to advance the profession toward the far-distant goal of perfection—to aid the solution of some of the problems and enigmas of life and organization—to add some material to the growing temple, whose foundations were so firmly laid by the Coan sage, and to do its part, as best it may, in the cause of humanity. Nor do I think that, so far, it has altogether failed. Many valuable contributions to science have been elicited—professional ambition has been stimulated—an *esprit-du-corps* has been successfully evoked and established.—the strength of the profession has acquired additional power by the union of its members. This association has been to physicians what the railroad and electric wires are to commerce, and the interchange of useful knowledge to states and nations. It has made us one, and, as I have just remarked, in unity there is power.

This association has stimulated thought. Chaotic and void would forever remain the masses of facts, accumulated by the observations of ages, but for the coordinating and logical power of reason. It sits in judgment on the silent phenomena, as a "refiner of fire, and a purifier of silver." It forces the voiceless facts to mount the tripod of the oracle, and speak forth words of wisdom. The scalpel, the crucible, the microscope, may be subsidiary to its purposes and ends, but they cannot supply its place. Fixed and patient thought, in medicine, as in other departments of science, is the Aladdin's lamp that lights the footsteps of the discoverer.—To stimulate attention and thought, is to accelerate many a new discovery—to hasten the advent and establishment of important principles yet in the womb of the future. May not our association do this more effectually than it has hitherto done?

Let all the contributions be read and attentively considered.—Such a course would certainly be more encouraging, as well as more respectful, to their authors. Let the reports be deliberately and fully discussed, and let them go forth to the world with the sanction or the criticisms of the association. This would require time, it is true, but if we have time to meet at all, surely a few days would make but little difference. The good that would be effected would yield a ten-fold compensation for the time employed. Every one must admit that three or four days is too short a time for the association readily to fulfil its annual mission.

I would, moreover respectfully suggest, that time be taken for the discussion of some of the leading topics of medical philosophy. Among these may be mentioned the nature, causes and treatment of cholera, yellow-fever, et cetera—Hygiene, and the laws of health affecting masses of men---Quarantine---the causes of mortality among children---the chemical and vital doctrines of life. Questions like these, indicated a year in advance for discussion, would excite a carefulness of investigation, and a degree of attention and thought which could not fail to clear away much of the darkness and doubt in which they are shrouded. Nothing so sharpens the intellectual powers as public debate. It fixes attention, and strains to the utmost every faculty. I have no hesitation in saying that facts enough have been accumulated to establish great and general principles, of which the medical world is yet in ignorance and doubt. Nothing would contribute more to demonstrate these principles than the collision of matured intellects in public debate.—What a mass of facts, and arguments, and demonstration, would be brought to bear, on any of the subjects alluded to, if some of the best minds in the profession would debate them, after a year's preparation! Observed facts are the crude materials of science—the intellect is the master builder of its august temple.

I make these considerations for your consideration. All the scientific meetings in this country and in Europe, employ more time than ours has hitherto employed. Evidently we must protract our sessions, if we would render them as serviceable to science as they may be. No member of the association will be required to remain longer than suits his wishes or convenience. Some fifty or sixty, more or less, would always be found to listen with eagerness to scientific papers, and engage with pleasure in scientific discussions.

The time has probably arrived for a change in our plan of organization, which will admit of the selection of a permanent place for the future meetings of the association. There are evident advantages incident to both the migratory and stationary plans.---These might, perhaps, be easily reconciled, and secured. A proposition, if I mistake not, was made some years ago, by the Smithsonian Institution, and I would respectfully suggest, whether it would not be in accordance with the best interests of the association, to hold biennial meetings in Washington, and the alternate ones, as now, at different points of our common country. We might thus secure all the advantages of a fixed abode, in the way of preserving the archives, making collections, etc., whilst by meeting in various localities, we could not fail to excite that wide-spread interest among the profession, and obtain such accessions of new members as would greatly enhance the high and useful objects of our association. Should this proposal meet with your approbation, I would further intimate, that policy would, perhaps, require the meetings of the association at the National Capital, to be held in the years of the short sessions of Congress.

I shall say but little of the legislative duties of the association. I shall say nothing of the propriety or impropriety of getting laws passed to regulate the practice of medicine, and furnish standards for candidates for the doctorate. Perhaps the association can do but little in this respect. Ours is a popular government, and the people are disposed to allow the largest freedom in everything pertaining to medicine, medical schools and physicians. Laws passed against quackery one year and revoked the next. Our country is the paradise of quacks. All good things have their attendant evils, and this unbridled liberty is one of the evils of a popular government. May we not hope, however, that even this evil may disappear, as general education and cultivation of the masses advance? At any rate the people are not disposed to put down the quacks, nor to require too high a degree of qualification for those of the regular profession. After all, laws can make only mediocre physicians. They can require the candidates to know only so much---to be qualified to a certain degree; and this degree will always be far lower than that to which the true lovers of knowledge would attain, without any legislation on the subject. The greater lights of the profession cannot be manufactured after any process of legislative enactment. Thirst of knowledge, self-love, philanthropy, burning ambition---these make the great physician and surgeon. These have made all the worthies of the past---not legislation. Legislation cannot drive the drone to the proud heights of professional eminence. When these heights are reached, it will be seen that the successful aspirant has been stimulated by a stronger power.

To him the laurel-blossoms of renown and the life-giving mission of his art, are dearer and more attractive than was the mystic bough of the sibyl to that eager Æneasor; than the golden apples, guarded by sleepless dragons to the Hesperian daughters.

Whatever course you may think proper to pursue, I am sure your objects will be, the advancement of science---the good of mankind---the honor and glory of the profession. We have the dignity and character of a noble calling to sustain---of a profession which has numbered, for two thousand years or more, some of the wisest and best of men in all countries and all times. It is no trivial matter, to sustain the rank and respectability of a vocation which can boast of a Hyppocrates---a Harvey---a Hunter---of the most erudite and beneficent of sages and philanthropists the world ever saw---of a profession which has furnished to every nation its *clarum et venerabile nomen.*

On the eve of the battle of the pyramids, Napoleon exclaimed---"Soldiers! from the height of yon monuments, forty centuries look down upon you." Gentlemen, from the heights of past ages, countless worthies of our God-like profession point and beckon to a goal more elevated than that which attracts legislators and conquerors, Solons and Cæsars.

On motion of Dr. J. Biddle, the thanks of the association were tendered to the President, Dr. Pope, for the eloquent and able address just delivered.

Dr. Hays then announced the various excursions planned by the committee of arrangements for the pleasure of the association.

Dr. Condie introduced a resolution on the subject of permanent membership, which was discussed, and an amendment offered by Dr. Watson, of New York. The whole subject was, on motion of Dr. White, of Buffalo, referred to a committee of three. The chairman appointed Drs. White, of Buffalo, Watson of New York, and Condie of Philadelphia, such committee.

A recess was then held, during which each State appointed a member of the nominating committee. Some amusement was created by the delegation from New York. A large number of its members had gathered upon one side of the hall, while a smaller party were in session in the center of the room, composed of some few of the older members of the profession. "Young America" yielded, and went over to the gray heads just in time to confirm their action, which had been duly arranged beforehand. This piece of generalship was received with a good-natured acquiescence.

On the resumption of business, invitations from Detroit, Nashville, and Chicago were presented, asking the association to meet in these localities next year.

Dr. D. D. Thompson, of Kentucky, moved that the regular order of business be dispensed with, to take up the amendments to the Constitution offered at the last annual meeting.

The association seemed to have had its dose of constitution, and manifested decided objections to any further tinkering with the organic law. This was a great blow to some of the prominent members, as it deprived them of their annual delivery of long speeches on a subject which is very interesting to them, if not to others.—we felt aggrieved, also, inasmuch as we intended to have had full and accurate reports of these abortive speeches, and we have based on them certain well-digested views of our own on the feasibility of pleasing every one.

A report was received through Dr. La Roche, from the committee on prizes. The report stated that the committee had received six essays in competition for the prize offered by the association. But, although these essays evinced much ability and extensive learning, but one was decided to possess those qualities which deserved the award of the prize. The essay was entitled "Statistics of Placenta Prævia." The name of the author was announced as Dr. James D. Trask, of White Plains, New York. (Applause.)

On motion, the report was accepted and the prize essay was referred to the committee on publication.

The committee on epidemics of Missouri, Iowa, Illinois, and Wisconsin, submitted a voluminous report, the abstract of which occupied a long time in reading.

On motion, the report was referred to the committee on publication.

Dr. White, from the committee to whom was referred the resolution in regard to the permanent members, submitted a report, recommending the adoption of the following resolution:

*Resolved*—That no permanent member who is not present at an annual meeting of this association, shall be required to pay the usual assessment; but no such permanent member shall be entitled to receive a copy of the proceedings of the meeting, unless by paying a sum equal to that assessed upon those who were present at such meeting.

The report of the committee was accepted and the resolution was adopted.

Dr. Sanford B. Hunt, of Buffalo, N. Y., from the committee on the hygrometrical state of the atmosphere in various localities, and its influence on health, submitted a report, pending the reading of which the hour of adjournment arrived, and the association adjourned to meet Wednesday morning at nine o'clock.

After the adjournment a large number of the members visited the Pennsylvania Hospital for the Insane.

SECOND DAY.

The association reassembled on Wednesday morning at nine o'clock.

Dr. J. Atlee, of Lancaster, addressed the convention on the subject of the stone made for the Washington National Monument, by J. A. Beck, a skillful American artist. Dr. A. stated that at a meeting of the association held in Richmond, Va., it was ordered that a stone be prepared for the monument. Dr. Pearson, of Salem, Mass., who advised that the representation of Hippocrates refusing presents, should be placed on the stone, but before he could consummate the plan the fearful accident happened at Norwalk, which swept him from their midst. It is said that Hippocrates, upon being sent for by Artaxerxes, King of Persia, to be the Court Physician, returned an answer, "tell your master I am rich enough; honor will not permit me to succor the enemies of Greece."

A resolution was adopted that no member be allowed to speak unless his name and residence be announced.

The committee on nominations, reported the following nominations:

*President*—GEORGE B. WOOD, Penn.

*Vice Presidents*—William M.———, Ala.; Daniel Tilden, Ohio; D. Humphrey Storer, Mass.; Grafton Tyler, D. C.

*Secretaries*---Francis West, Penn.; R. C. Foster, Tenn.

*Treasurer*---Caspar Wistar, Penn.

*Committee on Publication*---Francis G. Smith, Penn.

The President elect, on taking the chair, said that he felt deeply sensible of the honor conferred upon him, and though he had all his life devoted himself to the cause in which we are all engaged, yet he is unaccustomed to preside, and any errors which he may make, he hoped the association would excuse. He assured the convention that he would do his best to justify his appointment as presiding officer.

The committee further reported on the subject of the next place of meeiing, and recommended Nashville Tennessee.

A motion was made to substitute District of Columbia, in place of Nashville, Tennessee. Laid on the table.

Dr. A. B. Palmer, of Michigan, moved to substitute Detroit, Michigan, in place of Nashville.

A resolution was offered that the whole subject be referred to a special committee of five, to be appointed by the chair. Laid on the table.

The question was now taken, on the motion of Dr. Palmer, and it was adopted. Detroit, therefore, is the next place of meeting. The result was unanimously approved by rounds of applause.

Dr. R. C. Foster, appointed secretary, tendered his resignation, which was accepted.

Dr. Brodie, of Michigan, was appointed to fill the vacancy.

[Concluded in Number Six.]

